# Supplementary material for: Membrane-Supported Layered Coordination Polymer as an Advanced Sustainable Catalyst for Desulfurization
Source: Molecules. 2021 Apr 21;26(9):2404. doi: 10.3390/molecules26092404 (PMC8122353; doi:10.3390/molecules26092404)
Supplement: Supplementary file 1 [file molecules-26-02404-s001.zip › molecules-1151389-supplementary.pdf]

Supporting Information

Membrane-Supported Layered Coordination Polymer as an Advanced Sustainable Catalyst for Desulfurization

Fátima Mirante [1], Ricardo F. Mendes [2], Rui G. Faria [1], Luís Cunha-Silva [1], Filipe A. Almeida Paz [2] and Salete S. Balula [1]

[1] *REQUIMTE/LAQV & Department of Chemistry and Biochemistry, Faculty of Sciences, University of Porto, 4169-007 Porto, Portugal; fatimaisabelmirante@gmail.com; up201202396@fc.up.pt; l.cunha.silva@fc.up.pt*

[2] *CICECO - Aveiro Institute of Materials, Department of Chemistry, University of Aveiro, Campus Universitário de Santiago, 3810-193 Aveiro, Portugal; rfmendes@ua.pt*

* *Correspondence: filipe.paz@ua.pt (F.A.A.P.); sbalula@fc.up.pt (S.S.B);* rfmendes@ua.pt (R.F.M)

Membrane Characterization

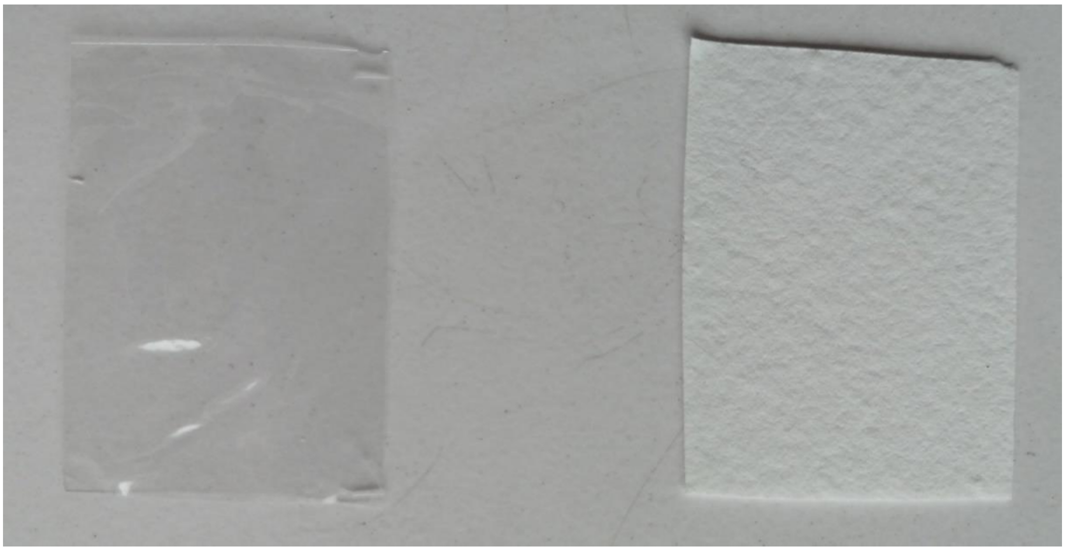

Figure S1. Picture of a (*left*) PMMA membrane and of the (*right*) UAV-59@PMMA composite membrane.

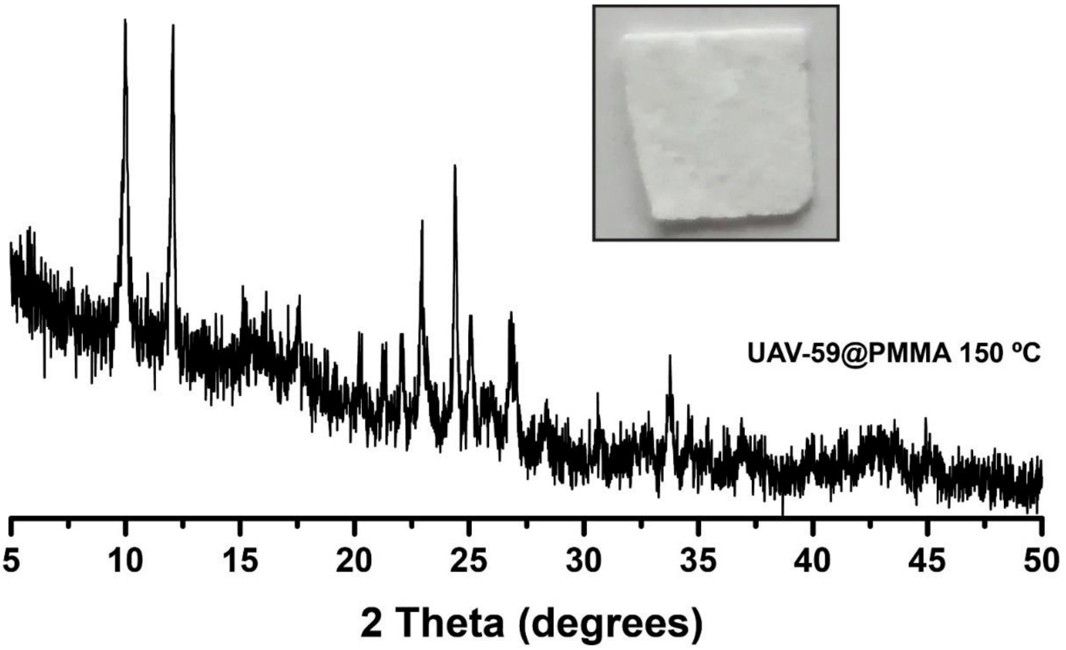

Figure S2. Powder X-ray diffraction patterns of the UAV-59@PMMA membrane after 24 h at 150 °C with the corresponding membrane picture on the top.

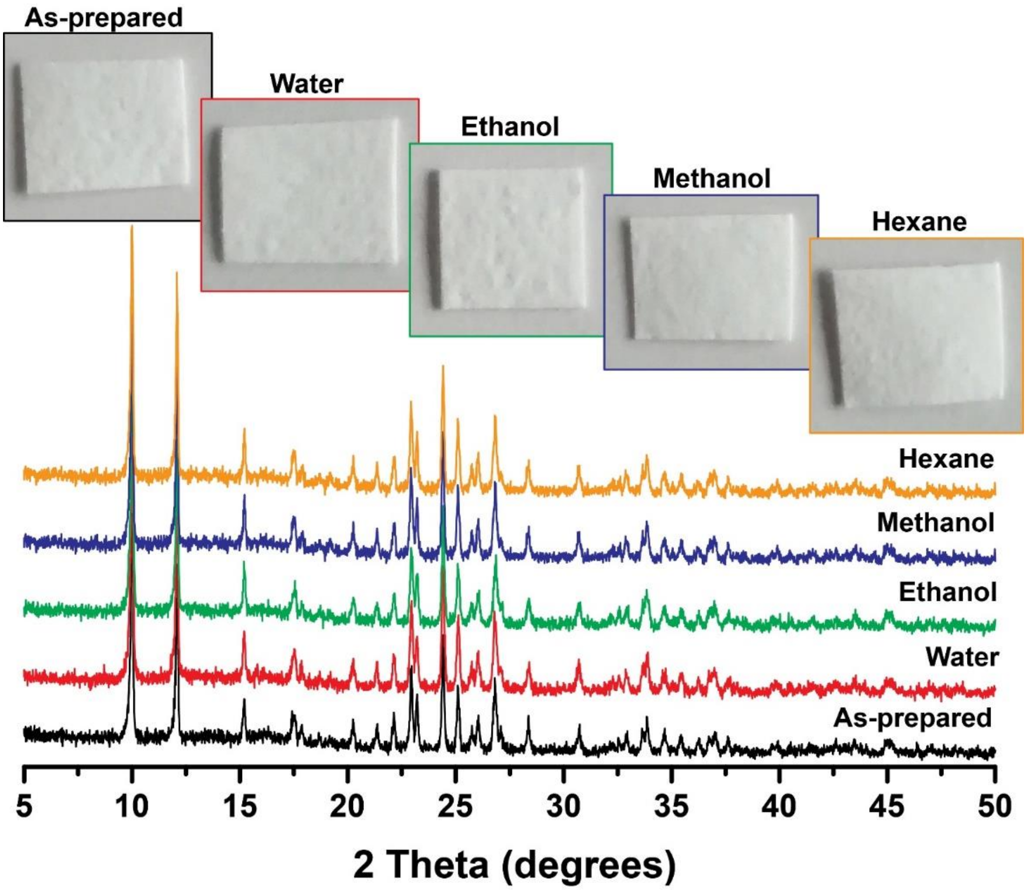

Figure S3. Powder X-ray diffraction patterns of the UAV-59@PMMA membranes (membrane pictures on the top) after 24 h immersed in different solvents.

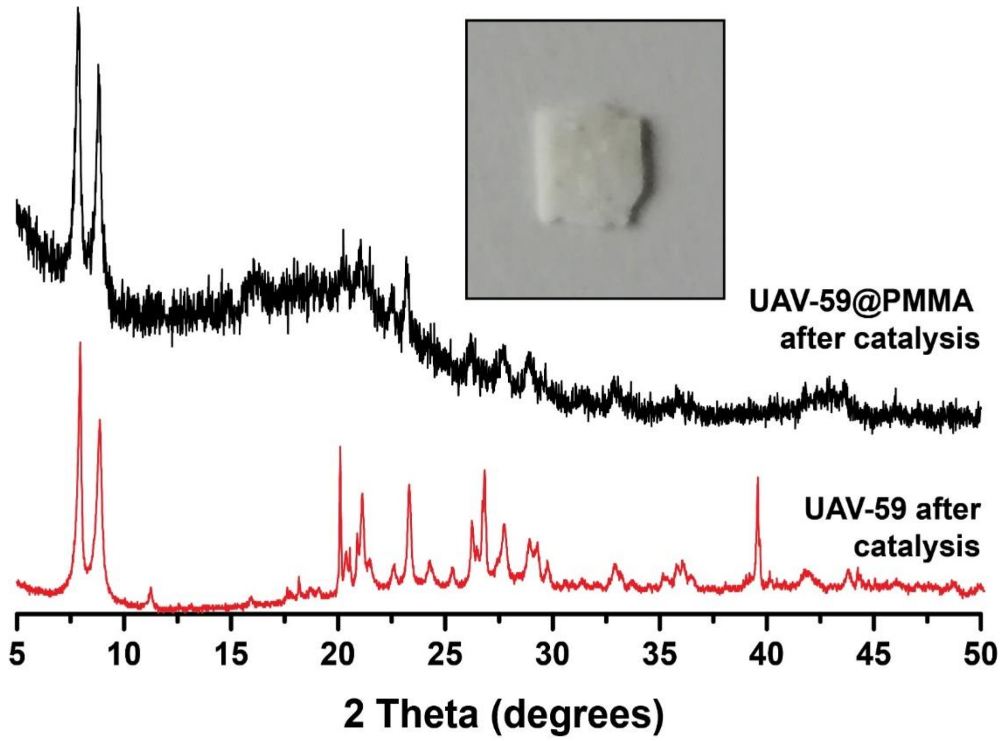

Figure S4. Comparison between powder X-ray diffraction patterns of the UAV-59 material in powdered form (**red**) and of the UAV-59@PMMA membrane (**black**) after catalysis. The inset picture pertains to the UAV-59@PMMA membrane after catalysis.
